# Long-Term Coffee Monoculture Alters Soil Chemical Properties and Microbial Communities

**DOI:** 10.1038/s41598-018-24537-2

**Published:** 2018-04-17

**Authors:** Qingyun Zhao, Wu Xiong, Yizhang Xing, Yan Sun, Xingjun Lin, Yunping Dong

**Affiliations:** Spice and Beverage Research Institute, Chinese Academy of Tropical Agricultural Sciences, Wanning, Hainan 571533 People’s Republic of China

## Abstract

Long-term monoculture severely inhibits coffee plant growth, decreases its yield and results in serious economic losses in China. Here, we selected four replanted coffee fields with 4, 18, 26 and 57 years of monoculture history in Hainan China to investigate the influence of continuous cropping on soil chemical properties and microbial communities. Results showed long-term monoculture decreased soil pH and organic matter content and increased soil EC. Soil bacterial and fungal richness decreased with continuous coffee cropping. Principal coordinate analysis suggested monoculture time was a major determinant of bacterial and fungal community structures. Relative abundances of bacterial *Proteobacteria*, *Bacteroidetes* and *Nitrospira* and fungal *Ascomycota* phyla decreased over time. At genus level, potentially beneficial microbes such as *Nitrospira* and *Trichoderma*, significantly declined over time and showed positive relationships with coffee plant growth in pots. In conclusion, continuous coffee cropping decreased soil pH, organic matter content, potentially beneficial microbes and increased soil EC, which might lead to the poor growth of coffee plants in pots and decline of coffee yields in fields. Thus, developing sustainable agriculture to improve soil pH, organic matter content, microbial activity and reduce the salt stress under continuous cropping system is important for coffee production in China.

## Introduction

Coffee is one of the most valuable agricultural export products from developing nations, and this industry is estimated to employ over 25 million people worldwide (Food and Agriculture Organization of the United Nations: http://www.fao.org)^1^. Coffee was introduced to China over 100 years ago. It is an important cash crop and was widely mono-cultured in the tropical and subtropical regions of south China, such as Yunnan and Hainan provinces^2^, with a total planting area of approximately 130,000 hectares. Coffee has been popularly used in the food and beverage industry because of its multiple pharmacological effects, such as reducing the risk of colorectal cancer^3^ as well as cardiovascular disease^4^. In China, the suitable regions for coffee planting are very limited, thus continuous monoculture coffee-growing regimes are commonly practiced to meet the increasing market demands. However, long-term monoculture often leads to poor growth, low yield and serious soil-borne disease in coffee plants^5,6^, which result in severe economic losses and hinder sustainable development of the coffee industry in China.

Continuous cropping usually inhibits plant growth, reduces yield and quality and weakens disease resistance in crops^7^. Continuous cropping obstacles, also known as replanted disease, are usually observed in agricultural crops (including both annual and perennial plants), such as soybeans^8^, melons^9^, bananas^10^ and apples^11^. Continuous cropping obstacles are generally attributed to the accumulation of autotoxic substances, deterioration of soil physiochemical properties, disturbance of the native soil microbiota or buildup of soil-borne pathogens^12,13^. A growing body of studies has confirmed that changes in the soil microbiota, such as increases in fungal pathogens, are responsible for the continuous cropping obstacles^14,15^. For example, Xiong *et al*.^16^ documented that the continuous cropping of black pepper resulted in a decrease in soil bacterial abundance and altered soil microbial community memberships and structures, which are in related to black pepper growth. Liu *et al*.^17^ demonstrated that soil overall bacterial communities were shaped by potato monoculture, and the soil-borne pathogen *Fusarium* was associated with disease incidence increase and yield decline. Hence, we hypothesize that continuous cropping may has a direct effect on soil microbial communities and chemical properties, in turn negatively impacting plant growth.

To best of our knowledge, the variations in soil microbial communities and soil chemical properties in coffee continuous cropping systems and their effects on coffee plant growth have not been reported. In this present study, we investigated both the bacterial and fungal communities in coffee-grown fields with 4, 18, 26 and 57 years of continuous cropping history in Hainan province China, using Illumina MiSeq technology^18^. In addition, we used a pot experiment with controlled conditions to evaluate the influence of soil from the fields that had undergone continuous cropping for different numbers of years on coffee plant growth. The aims of our study were to (1) assess the soil chemical characteristics and the overall soil microbial communities, including the bacterial and fungal diversity and compositional and structural changes associated with the different continuous cropping histories of the coffee fields, and (2) explore the underlying relationships between the dominant soil microbial taxa, chemical characteristics and coffee plant growth in pots with controlled conditions.

## Results

### Plant Dry Weight from the Time-Series Pot Experiment and Fresh Weight of Coffee Fruit in the Fields

**Figure 1:**
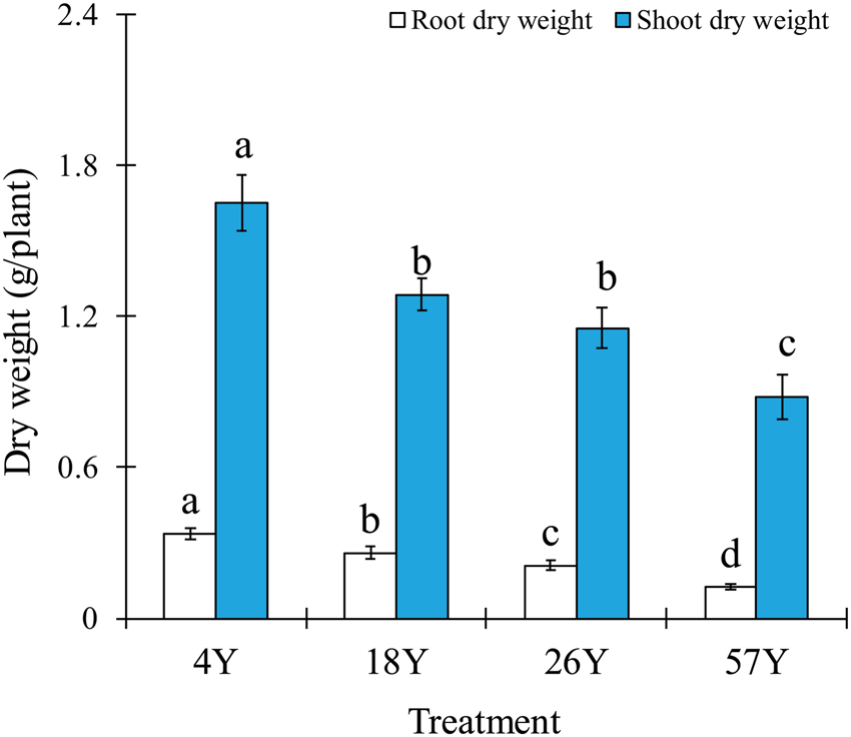
Plant dry weight of coffee seedlings in pot experiments. Vertical bars represent the mean ± standard deviation of three replicates. Bars with different letters indicate significant differences (*P* < 0.05) based on Duncan’s test. “4Y”, “18Y”, “26Y”, and “57Y” stand for 4 coffee fields with 4, 18, 26 and 57 years of continuous cropping history, respectively.

In the coffee pot experiment, plant dry weight significantly decreased along the time-series of field soils (*P* < 0.05; Duncan’s test, Fig. 1). The “4Y” plantation soil showed the highest shoot and root dry weight, while the “57Y” plantation soil resulted in the lowest shoot and root yield, with the shoot and root dry weight from the “57Y” samples decreasing by 47% and 65%, respectively, compared with the “4Y” plantation soil. In addition, we found that fresh weight of coffee fruit also significantly decreased along the time-series of field soils (*P* < 0.05; Duncan’s test, Table S2).

### The Chemical Characteristics of Soils from Four Time-Series Fields Experiments

As shown in Table 1, the soil electrical conductivity (EC), available P, Fe and Zn contents increased with the continuous cropping of coffee. The “4Y” plantation soil showed the highest organic matter (OM) content with lowest Mg content, while OM showed no significant difference among the other three continuous cropping soils (“18Y”, “26Y” and “57Y”). Soil pH significantly declined from 6.3 (“4Y”) to 4.5 (“26Y”), and it showed a slight increase in the “57Y” continuous cropping soil. The “26Y” field soil revealed the highest contents of soil available N, available K and B.

**Table 1:** Soil characteristics from the four time-series coffee fields.

Coffee fields	pH	EC (electrical conductivity) (μs/cm)	OM (organic matter) (g/kg soil)	Available N (mg/kg soil)	Available P (mg/kg soil)	Available K (mg/kg soil)	
4Y	6.30 ± 0.09a	37.60 ± 1.23d	22.91 ± 1.28a	52.16 ± 2.49d	39.63 ± 1.65d	108.21 ± 6.37b	
18Y	5.48 ± 0.12b	71.15 ± 3.64c	18.30 ± 0.97b	57.86 ± 1.92c	53.61 ± 4.16c	93.76 ± 2.31c	
26Y	4.53 ± 0.17c	76.36 ± 2.01b	18.72 ± 1.41b	79.51 ± 5.36a	146.18 ± 11.23b	169.2 ± 4.50a	
57Y	5.41 ± 0.23b	88.85 ± 5.39a	17.16 ± 0.83b	66.39 ± 3.72b	214.10 ± 9.51a	79.6 ± 2.02d	
**Coffee fields**	**Ca** **(mg/kg soil)**	**Mg** **(mg/kg soil)**	**B** **(mg/kg soil)**	**Fe** **(mg/kg soil)**	**Mn** **(mg/kg soil)**	**Cu** **(mg/kg soil)**	**Zn (mg/kg soil)**
4Y	82.30 ± 8.12b	29.12 ± 1.03c	0.39 ± 0.02a	15.95 ± 0.93c	22.41 ± 1.35c	0.92 ± 0.02b	1.56 ± 0.10c
18Y	103.91 ± 7.22a	41.02 ± 3.51a	0.28 ± 0.01b	18.09 ± 0.83b	35.05 ± 0.87a	0.73 ± 0.03c	1.63 ± 0.04c
26Y	98.05 ± 5.90a	34.56 ± 2.49b	0.43 ± 0.02a	20.01 ± 1.50ab	29.74 ± 1.01b	1.61 ± 0.09a	2.02 ± 0.12b
57Y	71.44 ± 5.36c	37.49 ± 2.72ab	0.41 ± 0.03a	22.91 ± 1.07a	31.93 ± 2.78b	1.52 ± 0.04a	2.39 ± 0.09a

Data are expressed as means ± standard wdeviation (n = 3). Data in a column with different letters differ significantly (*P* < 0.05) based on Duncan’s test.

### Bacterial and Fungal α-Diversity

The sobs (the number of observed OTUs), Chao1 and ACE indexes of both the bacterial and fungal communities significantly decreased after 26 years of coffee cropping (*P* < 0.05; Duncan’s test, Table 2), while the Shannon index for bacteria showed no obvious difference among the four time-series coffee fields. In addition, the fungal Shannon index value from the 26-year coffee field was significantly lower (*P* < 0.05; Duncan’s test, Table 2) than those from the 4-year and 18-year fields, while there were no significant difference in the fungal Shannon index between the 4-year field and 57-year field, and between the 26-year field and 57-year field. Rarefaction curves of the mean number of sequences for the 4 replicates from each field were calculated (Figure S2), results show that the number of observed OTUs in the bacterial and fungal communities significantly decreased with an increase in coffee planting years (*P* < 0.05; Duncan’s test, Table 2).

**Table 2:** Bacterial and fungal alpha-diversity indexes of soil from four time-series coffee fields.

Microbial community	Coffee fields	Sobs	Chao1	ACE	Shannon
Bacteria	4 Y	2344.47 ± 76.59a	3952.57 ± 72.85a	5125.67 ± 88.86a	6.95 ± 0.08a
	18 Y	2141.09 ± 166.90ab	3650.85 ± 298.77ab	4732.01 ± 395.28ab	6.65 ± 0.48a
	26 Y	2044.46 ± 155.71b	3608.94 ± 199.12ab	4928.19 ± 255.20ab	6.57 ± 0.17a
	57 Y	1918.37 ± 143.89b	3336.96 ± 78.23b	4467.34 ± 340.24b	6.48 ± 0.30a
Fungi	4 Y	663.30 ± 11.33a	954.84 ± 36.30a	1027.06 ± 42.04a	4.21 ± 0.17a
	18 Y	627.24 ± 22.29ab	914.39 ± 37.42ab	979.69 ± 55.37ab	4.27 ± 0.23a
	26 Y	584.01 ± 32.87b	856.74 ± 43.34b	939.94 ± 58.83b	3.87 ± 0.17b
	57 Y	590.63 ± 43.14b	854.61 ± 56.63b	937.07 ± 44.57b	4.05 ± 0.22ab

Data were expressed as mean ± standard deviation (n = 3). The data in a column with a different letter differ significantly at Duncan’s significance level 0.05.

### Bacterial and Fungal β-Diversity

**Figure 2:**
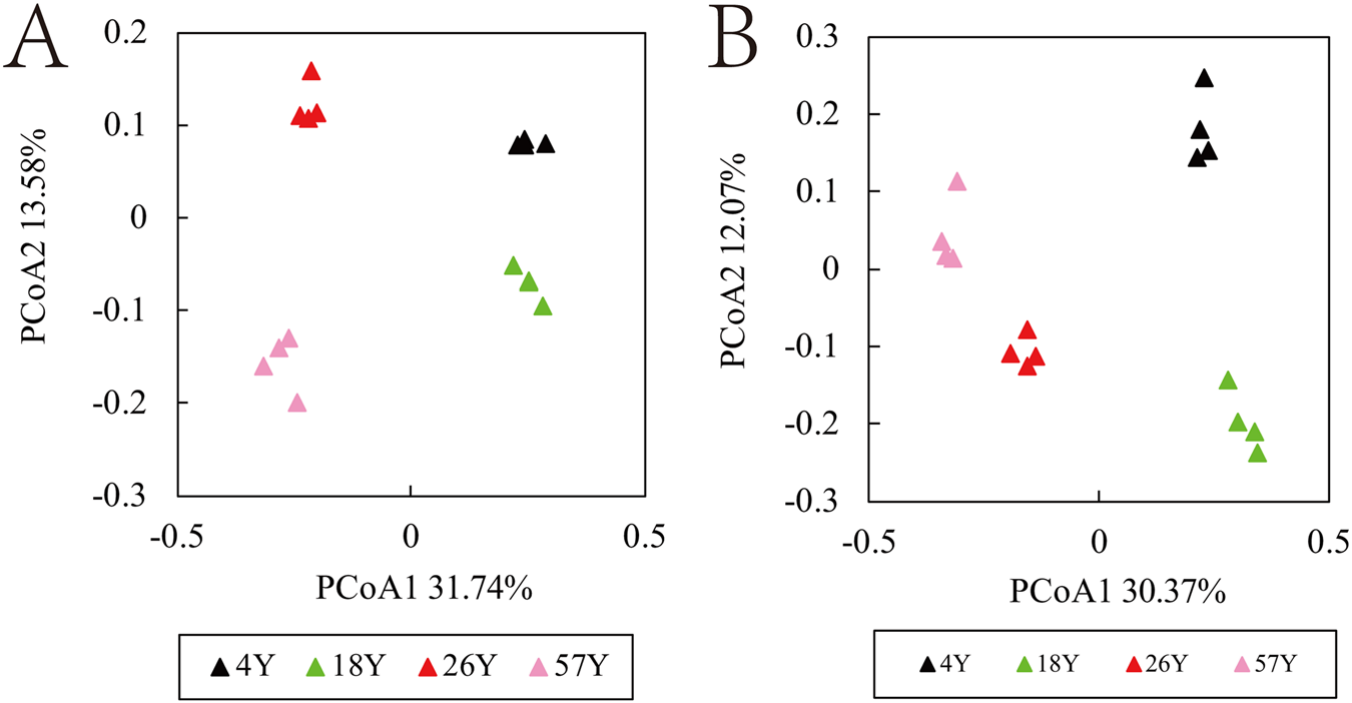
PCoA plots of bacterial (panel A) and fungal (panel B) communities in a time-series of coffee fields continuously cropped for different numbers of years. “4Y”, “18Y”, “26Y”, and “57Y” represent fields continuously cropped for 4, 18, 26, or 57 years, respectively.

Bray-Curtis dissimilarity principal coordinate analysis (PCoA) indicated distinct patterns in both the bacterial and fungal communities in the four time-series coffee fields, with the first two axes explaining 45.32% of the total variation in the bacterial data and 42.44% of the variation in the fungal data, respectively (Fig. 2). In addition, both the bacterial and fungal communities in the 4-year (4Y) soil sample and 18-year (18Y) soil sample were well separated from the 26Y and 57Y soil samples along the first component (PCoA1).

### Bacterial and Fungal Community Composition

**Figure 3:**
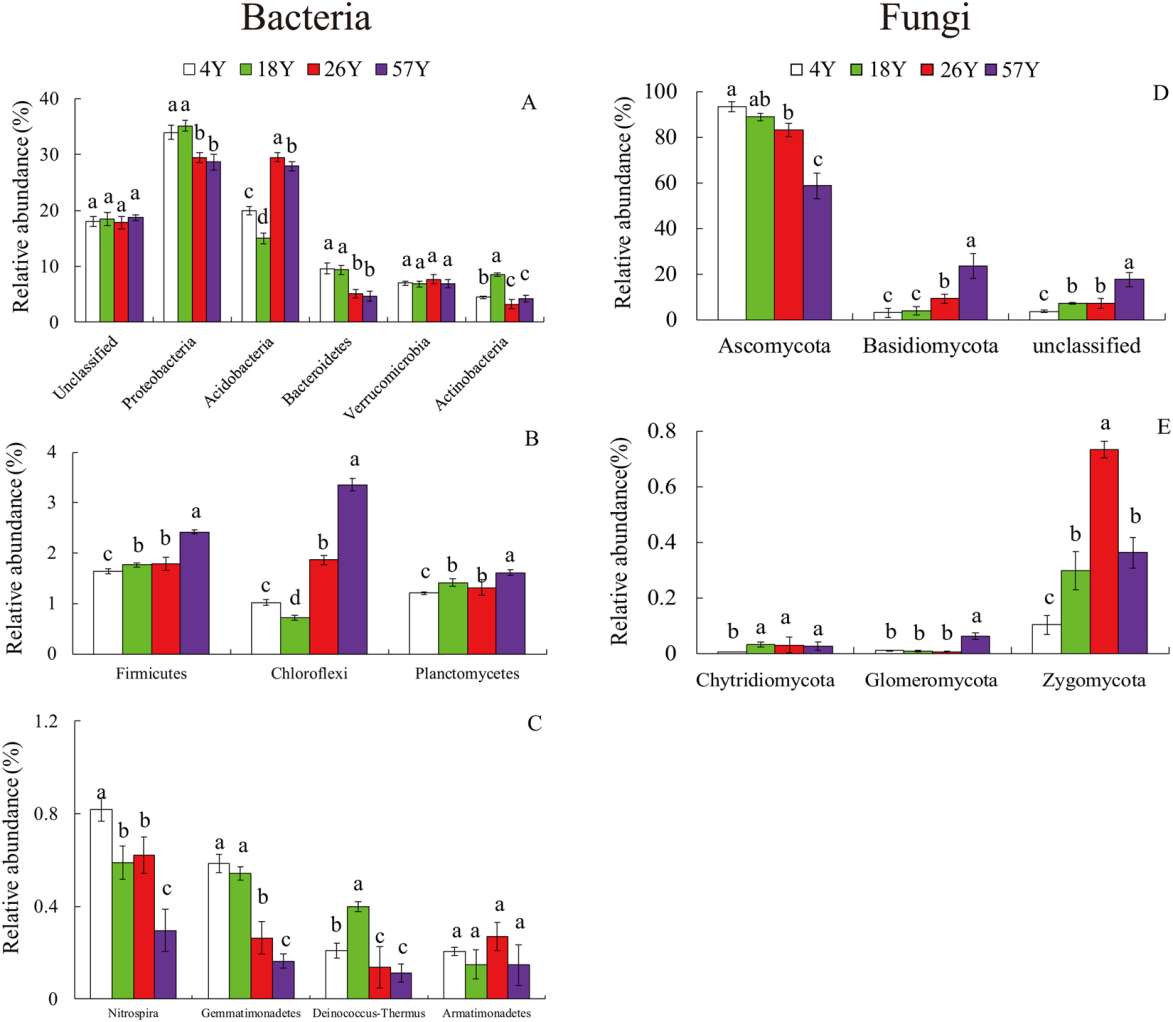
Relative abundances of the main bacterial (panel: A, B, C) and fungal (panel: D, E) phyla over a time-series of coffee fields continuously cropped for different numbers of years. Vertical bars represent the mean ± standard deviation of four replicates. Bars with different letters indicate significant differences (*P* < 0.05) based on Duncan’s test. “4Y”, “18Y”, “26Y”, and “57Y” represent fields with 4, 18, 26, or 57 years of continuous cropping, respectively.

The dominant phyla in the bacterial and fungal communities varied greatly among the different soil samples (Fig. 3). Twelve bacterial phyla were identified in the time-series coffee fields (Fig. 3A–C). Among these phyla, *Proteobacteria* corresponded with 31.82% of the total bacterial sequences (average relative abundance across all the samples), followed by *Acidobacteria* (23.07%), *Bacteroidetes* (7.20%), *Verrucomicrobia* (7.08%), *Actinobacteria* (5.13%), *Firmicutes* (1.90%), *Chloroflexi* (1.74%) and *Planctomycetes* (1.39%); these were the dominant bacterial phyla (relative abundance > 1%). Five fungal phyla, *Ascomycota* (80.95%), *Basidiomycota* (9.83%), *Chytridiomycota* (0.02%), *Glomeromycota* (0.02%), *Zygomycota* (0.37%), and unclassified fungi (8.59%) were detected in all soil samples (Fig. 3D,E). In addition, the relative abundances of the bacterial *Proteobacteria*, *Bacteroidetes*, *Nitrospira* and *Gemmatimonadetes* phyla and the fungal *Ascomycota* phylum decreased with an increase in years of continuous cropping, whereas the bacterial *Firmicutes* and fungal *Basidiomycota* phyla increased over the time series.

**Figure 4:**
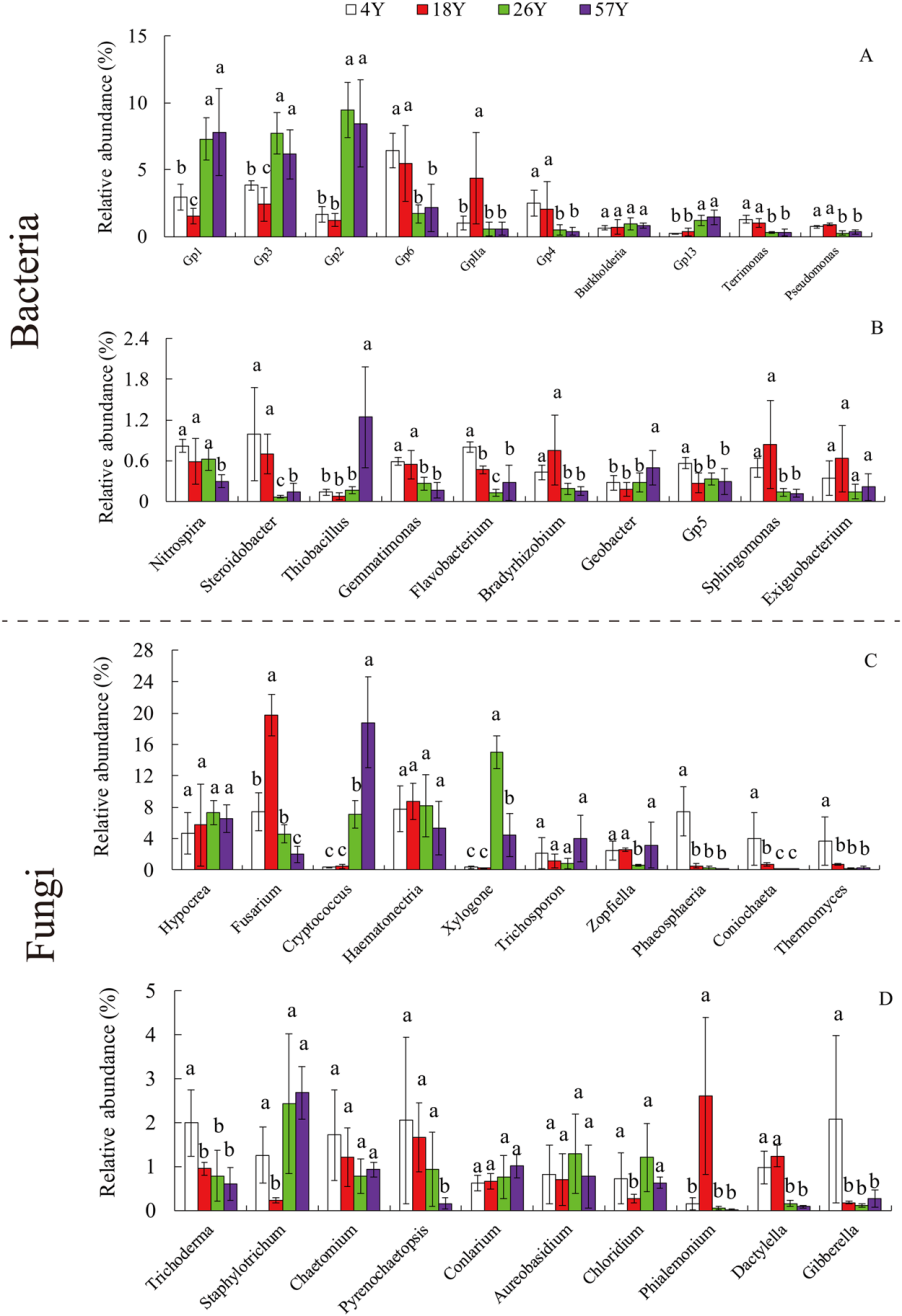
Relative abundances of the top 20 bacterial (panel A: top 10 genera; panel B: top 11–20 genera) and fungal genera (panel C: top 10 genera; panel D: top 11–20 genera) in the 4 replanted coffee fields. Vertical bars represent the mean ± standard deviation of four replicates. Bars with different letters indicate significant differences (*P* < 0.05) based on Duncan’s test. “4Y”, “18Y”, “26Y”, and “57Y” represent fields with 4, 18, 26, or 57 years of continuous cropping, respectively.

Furthermore, the 20 most abundant genera across the four time-series coffee fields were investigated (Fig. 4), and the relative abundances of the bacterial *Gp6*, *Gp4*, *Terrimonas*, *Pseudomonas*, *Nitrospira* and *Gemmatimonas*, and the fungal *Phaeosphaeria*, *Coniochaeta*, *Thermomyces* and *Trichoderma* significantly declined with the continuous cropping of coffee. In contrast, the relative abundance of bacterial *Thiobacillus* and fungal *Cryptococcus* significantly increased in the 57 years coffee fields.

### Effects of Soil Chemical Variables on Abundant Phyla

**Figure 5:**
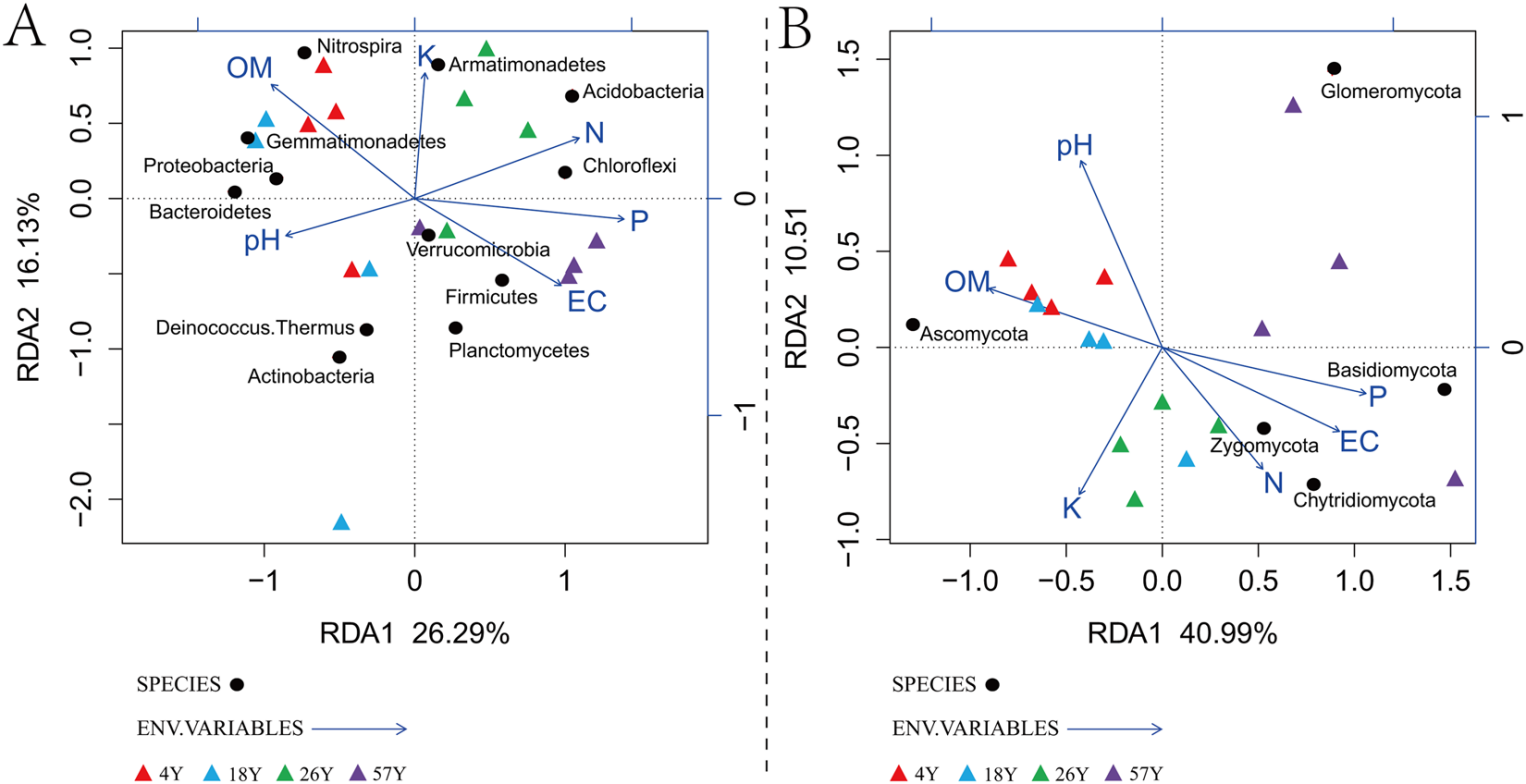
Redundancy analysis (RDA) based on the relative abundances of bacterial (panel A) and fungal (panel B) phyla and environmental variables (pH, EC, OM, Available N, P, and K) for individual samples collected from the 4 replanted coffee fields. “4Y”, “18Y”, “26Y”, and “57Y” stand for 4 coffee fields with 4, 18, 26 and 57 years of continuous cropping history, respectively.

The Redundancy analysis (RDA) results indicate that soil pH, organic matter (OM), EC, and available N, P and K contents explained 42.42% and 51.50% of the total variation in the bacterial and fungal communities, respectively (Fig. 5A,B). For bacteria, the 4Y and 18Y samples were clearly separated from the 26Y and 57Y samples by the first component (RDA1), and the 26Y treatment was separated from the 57Y treatment by the second component (RDA2). *Proteobacteria*, *Nitrospira* and *Bacteroidetes* were prevalent in the 4Y and 18Y samples and were more associated with soil OM, while *Acidobacteria* and *Firmicutes* were more related to soil available N and EC, respectively. For fungi, the 57Y samples were clearly separated from the 4Y, 18Y and 26Y samples by the first component (RDA1), and 4Y and 18Y were more related to soil pH and organic matter. *Ascomycota* was more associated with soil OM, while *Basidiomycota* was highly prevalent in the 57Y samples, which was linked to soil P and EC.

### Pearson Correlation Coefficients between Soil Chemical Variables, Abundant Bacterial and Fungal Phyla and Genera, and Plant Growth in Pots and Fresh Weight of Coffee Fruit in the Fields

As shown in Table 3 and Table S4, soil EC, available P, Fe and Zn content showed significant (*P* < 0.05) negative correlations with the coffee plant dry weight in pots and fresh weight of coffee fruit in the fields. Soil OM content was positively correlated with coffee plant shoot dry weight in pots. We found a significant (*P* < 0.05) negative correlation between bacterial *Firmicutes*, fungal *Basidiomycota* and plant root dry weight, while *Ascomycota* positively correlated with plant root dry weight. The relative abundance of *Proteobacteria* and *Bacteroidetes* were significantly (*P* < 0.05) and positively correlated with fresh weight of coffee fruit in the fields. Moreover, we found that the relative abundance of bacterial Gp4, Gp13, *Gemmatimonas* and *Nitrospira* were significantly (*P* < 0.05) correlated with coffee plant root dry weight. Gp4 and Gp6 showed significantly (*P* < 0.05) and positively correlated with fresh weight of coffee fruit in the fields. In terms of fungi, significant (*P* < 0.05) positive correlations between *Coniochaeta* and *Trichoderma* with coffee plant shoot dry weight were observed.

**Table 3:** Pearson’s correlation relationships between soil properties, bacterial and fungal abundant phyla (RA > 1%), abundant genera (top 20) and plant dry weight (root and shoot dry weight) in pots.

Soil properties	Root dry weight	Shoot dry weight	**Bacterial and fungal phyla**	Root dry weight	Shoot dry weight	**Bacterial abundant genera**	Root dry weight	Shoot dry weight	**Fungal abundant genera**	Root dry weight	Shoot dry weight
pH	0.587	0.638	***Proteobacteria***	0.824	0.766	***Gp1***	−0.793	−0.734	***Hypocrea***	−0.774	−0.8
EC	−0.943*	−0.978*	***Acidobacteria***	−0.666	−0.603	***Gp2***	−0.792	−0.757	***Fusarium***	0.471	0.364
OM	0.882	0.935*	***Bacteroidetes***	0.89	0.856	***Gp3***	−0.597	−0.549	***Cryptococcus***	−0.928*	−0.873
N	−0.63	−0.644	***Verrucomicrobia***	−0.08	−0.082	***Gp6***	0.864	0.853	***Haematonectria***	0.704	0.616
P	−0.955*	−0.914*	***Actinobacteria***	0.26	0.163	***GpIIa***	0.305	0.195	***Xylogone***	−0.421	−0.422
K	0.128	0.113	***Firmicutes***	−0.907*	−0.866	***Gp4***	0.910*	0.893	***Trichosporon***	−0.514	−0.426
Ca	0.359	0.243	***Chloroflexi***	−0.889	−0.822	***Burkholderia***	−0.689	−0.703	***Zopfiella***	−0.106	−0.077
Mg	−0.525	−0.621	***Planctomycetes***	−0.888	−0.892	***Gp13***	−0.923*	−0.889	***Phaeosphaeria***	0.807	0.876
B	−0.339	−0.242				***Terrimonas***	0.900*	0.892	***Coniochaeta***	0.843	0.901*
Fe	−0.998**	−0.983**	***Ascomycota***	0.938*	0.894	***Pseudomonas***	0.704	0.649	***Thermomyces***	0.828	0.89
Mn	−0.593	−0.689	***Basidiomycota***	−0.924*	−0.87	***Nitrospira***	0.954*	0.951*	***Trichoderma***	0.895	0.944*
Cu	−0.737	−0.686				***Steroidobacter***	0.888	0.888	***Staphylotrichum***	−0.692	−0.612
Zn	−0.960*	−0.917*				***Thiobacillus***	−0.817	−0.753	***Chaetomium***	0.839	0.874
						***Gemmatimonas***	0.938*	0.904*	***Pyrenochaetopsis***	0.985*	0.956*
						***Flavobacterium***	0.814	0.848	***Conlarium***	−0.952*	−0.910*
						***Bradyrhizobium***	0.594	0.505	***Aureobasidium***	−0.114	−0.11
						***Geobacter***	−0.758	−0.669	***Chloridium***	−0.122	−0.073
						***Gp5***	0.751	0.83	***Phialemonium***	0.25	0.136
						***Sphingomonas***	0.638	0.558	***Dactylella***	0.789	0.73
						***Exiguobacterium***	0.452	0.365	***Gibberella***	0.746	0.824

RA is relative abundance. *Represent significance at *P* < 0.05 and **represent significance at *P* < 0.01.

## Discussion

Long-term monocultures of coffee have led to severe declines in coffee yields (Table S2) and plant biomass in the fields, which have been commonly observed in Hainan province, China^5,6^. In the present study, we confirmed that continuous cropping problems exist in the coffee cropping system, with our pot experiments showing that the coffee shoot and root dry weights significantly decreased with increasing years of monoculture (Fig. 1). These results correspond with those of Xiong *et al*.^16^, who reported that the dry weight of black pepper shoots significantly decreased under black pepper continuous cropping system. This general phenomenon has also been found in other crops, such as *Angelica sinensis*^19^, cucumber^20^ and soybean^21^, the growth of which was severely inhibited in continuous cropping agro-ecological systems.

Soil chemical characteristics, such as pH and organic matter, are widely recognized as being central to the sustainability of agricultural production systems. Soil pH and organic matter content decreased with the continuous cropping of coffee (Table 1). The long-term application of chemical fertilizers and lack of organic materials returning to the soil might be the main factors resulting in declines of soil pH and organic matter in coffee field^22^. Another possible cause of soil acidification in coffee cropping systems is the accumulation of allelochemicals^23^ or coffee residue^24^. The results of this study opens up future research directions for understanding the relationship between coffee plant allelochemicals or residue with soil pH. Moreover, some studies showed that extracts of coffee residue or allelochemicals exhibiting the ability to inhibit seed germination and root growth of some plant species^24^. In contrast, soil P content significantly increased with the coffee monoculture, which may be attributed to overuse of chemical fertilizer^16,17^ including Calcium superphosphate in this study (Table S1) or the field coffee plants under long-term monoculture can’t utilize soil available P efficiently^25^. Thus, it is necessary to test the available P transformation from soil to coffee plant and the function with coffee plant growth in our future study. In addition, we found soil EC, available P, Fe and Zn were increased with the long term continuous coffee cropping and showed significant (*P* < 0.05) negative correlations with the coffee plant dry weight in pots and fresh weight of coffee fruit in the fields (Tables 1, 3 and S4), which suggested that salt stress or heavy metal stresses under the long-term continuous cropping system maybe one possible reason for the poor growth of coffee plant in pots and declined yields of fruit coffee in the fields. In sum, the decreased soil pH and organic matter, increased salt stress, or accumulated coffee residue and allelochemicals under long-term coffee monoculture may attributed the poor growth of the coffee plants in pots and low yields of coffee beans in the fields.

Many studies indicated that soil microbial communities play a major role in soil functions and ecosystem sustainability^26,27^. Unveiling soil microbial community variations under continuous cropping system is helpful in understanding the poor growth of coffee associated with long-term monoculture. In the present study, soil bacterial and fungal richness decreased over the years of coffee cropping. These results were consistent with those of some previous studies. For example, bacterial diversity significantly decreased after 55 years of black pepper continuous cropping^16^. Tan *et al*.^28^ documented that fungal diversity and abundance decreased with increasing years of the continuous cropping of *Panax notoginseng* in root-rot diseased soil. Liu *et al*.^17^ noted that bacterial diversity and richness indexes significantly and linearly decreased with an increase in monoculture years. Loss of microbial diversity is considered to be a major threat to ecosystem functions^29^. Thus, reductions in soil fungal and bacterial diversity are linked with the partial loss of soil functions, such as disease suppression or plant growth promotion^30,31^ and might have resulted in the poor coffee growth in continuous monoculture systems.

The PCoA results in this study indicated that the soil bacterial and fungal communities were influenced by long-term continuous cropping (Fig. 2). A similar result was reported by Xiong *et al*.^14^, in which the duration of vanilla monoculture had a significant effect on the variation in bacterial and fungal community structures. This phenomenon was also detected in *Panax notoginseng*^28^ and peanut^32^ continuous cropping systems. Moreover, PCoA analysis showed that the bacterial and fungal communities differed after 26 years continuous cropping. Combined with the RDA results (Fig. 5), we can infer that the changes in soil chemical properties could have contributed to the distinct variations in bacterial and fungal community structures, as soil chemical properties play critical roles in microbial community structures^33,34^. It is also worth noting that microbial changes in the long-term coffee monoculture systems are not only due to the variations in soil chemical properties but might also be caused by the long-term effects of coffee plant residues or root exudates^5,35^.

The long-term continuous cropping of coffee had a significant effect on the bacterial and fungal community compositions. For bacteria, the relative abundances of *Proteobacteria*, *Bacteroidetes* and *Nitrospira* decreased with the long-term continuous cropping of coffee (Fig. 3). Mendes *et al*.^36^ identified *Proteobacteria* as the most dynamic taxon associated with *Rhizoctonia* disease suppression. *Bacteroidetes* was recognized as an important indicator of plant heath in vanilla^14^, black pepper^16^ and banana^37^ cropping systems. *Nitrospira* (*Nitrospirae*) are known for their activities in nitrite oxidizing, which impacts soil N cycling^38,39^. In addition, we found that the relative abundance of *Nitrospira* showed a significant (*P* < 0.05) positive correlation with coffee plant growth in pots (Table 3). At the genus level, *Gp6*, *Gp4*, *Pseudomonas* and *Gemmatimonas* significantly declined with the continuous cropping of coffee (Fig. 4). *Pseudomonas* has been popularly reported as containing active antagonistic microbes that protect plants from attack from pathogens such as *Rhizoctonia solani*^40^, *Fusarium oxysporum*^41^ and *Phytophthora infestans*^42^. Moreover, *Gp4*, *Gp6* and *Pseudomonas* were found to be indicators of *Fusarium* wilt disease-suppression in banana cropping systems^37^. Interestingly, we found soil pH showed positive relation with coffee plant growth in pots and fresh weight of coffee fruit in the field (Table 3 and S4), but not significantly. While, previous researches showed that Gp4 and Gp6 were positively correlated with soil pH^43,44^ and were easily adjusted with soil pH change, we speculated that the decline of some potentially beneficial microbes induced by the declined soil pH under long-term coffee monoculture may be one determined factor for coffee poor growth in pots and low yield in the fields. Future studies utilizing different amounts of chemical fertilizer or regulating different value of soil pH in pots experiments would be helpful for disentangling the relationships between soil chemical properties or soil pH, the changes of beneficial microbes and coffee growth. *Gemmatimonas* significantly decreased with the continuous coffee cropping and showed a significantly (*P* < 0.05) positive correlation with coffee plant biomass in pots, which was in line with Yang *et al*.^45^. They found that *Gemmatimonas* (Gemmatimonadetes) showed positive relationships with the dry weight of wheat shoot. In contrast, the relative abundance of *Thiobacillus* significantly increased in the 57 years coffee fields, *Thiobacillus* are obligate autotrophs using elementary sulfur, thiosulfate or polythionates as energy sources, which tolerate organic compounds. The high relative abundance of *Thiobacillus* in the 57 years coffee fields might be induced by the anaerobic conditions because of soil compaction under long-term continuous cropping, the long-term application of organic fertilizer^46^ or the coffee plant residue, but we need a future study to test such a role. Among the fungi, *Trichoderma* significantly declined with the long-term continuous cropping of coffee, and this genus showed a significant (*P* < 0.05) positive correlation with coffee plant shoot dry weight in pots (Table 3). The genus *Trichoderma* has been recognized for its plant growth-promoting and biocontrol abilities^47^. The decrease in potential beneficial microbes might be attributed to the poor growth of coffee plants in long-term monoculture systems^6^, and further studies identifying the potential beneficial microbes in coffee cropping fields, including bacterial *Gemmatimonas*, *Nitrospira* and *Pseudomonas* and fungal *Trichoderma* isolates with effective plant growth promoting abilities, are needed.

In conclusion, this is the first report describing the responses of bacterial and fungal communities to coffee long-term monoculture. Our results reveal that the poor growth of coffee plant in pots and declined yields of coffee fruit in the fields under the long-term continuous cropping system can be linked to the shifts in soil chemical properties and microbial communities, such as decreases in soil organic matter and reductions in microbial diversity and potentially beneficial microbes. Future studies are needed to further disentangle the triangle relationships between soil characteristics, potentially beneficial microbiota and coffee growth. This study gives us a valuable avenue for developing sustainable agricultural measures to improve microbial activity in promoting coffee growth in soils subjected to continuous cropping, which is crucial for coffee production in the tropical areas of China.

## Materials and Methods

### Sampling Site

The experimental site is located at the Spice and Beverage Research Institute, Chinese Academy of Tropical Agricultural Sciences, Xinglong, Hainan province, China (110°20′E, 18°73′N). The annual precipitation and mean annual temperature are 2201 mm and 24.5 °C, respectively. The soil type, agronomic management and fertilization regimes (please see Table S1) were consistent in the different continuous cropping years of coffee fields. Soil samples were collected from four coffee fields that were continuously cropped for 4, 18, 26 and 57 years (marked as “4Y”, “18Y”, “26Y” and “57Y”, respectively) in April, 2016 (each field with the size around 4 to 5 mu, 1 mu = 667 m^2^; around 100 plants per mu). The researchers started to plant coffee in the fields close to the Spice and Beverage Research Institute 57 years ago, there were no independent replicates in the fields. Thus, in order to adequately represent each time-series coffee cropping field, we randomly chosen 4 subplots with 12 coffee plants in each coffee field to generate 4 replicates, and randomly collected 15 cores (0–30 cm in depth, 2.5 cm in diameter) from each subplot and mixed thoroughly to compose one sample, then, we used the 4 subplots as the 4 replicated for each time-series coffee field (map with the detailed sampling regime was provided in the Figure S1). Fresh weight of coffee fruit for the 4 time-series coffee fields were calculated based on the harvested fresh coffee beans during the last year of the 12 coffee plants in each subplot (Table S2). All 16 soil samples were placed separately into sterile plastic bags and transported to the laboratory in an ice box. After passing through a 2 mm sieve, one part of each sample was stored at −80 °C for DNA extraction, and the remainder was air dried for the analysis of soil characteristics.

### Pot Experiment Description

We used pot experiment under controlled conditions to evaluate the influence of soil chemical properties and already established microbial communities from the four time-series coffee fields on coffee plant growth from April to June, 2016. For the pot experiment, soil was collected with a shovel in the direct vicinity of the cores used for DNA extraction, we randomly obtained soil from three subplots (three replicates) within each coffee field. We therefore had three independent blocks (i.e., the three replicates) for the four treatments (i.e., four time-series of coffee field soils), with each block containing seven pots. Each pot contained 3.0 kg of soil, and uniform coffee seedlings with 2 leaves (one year old) were transplanted into each pot, and irrigation was performed weekly to keep the soil moisture constant during the next two months before harvest. In order to reduce the accident error between the 7 plants in pots for each replicate (*i*.*e*., we excluded outlier coffee plants among the 7 coffee plants), we sampled the four looks similar plants (based on their growth performance) from the 7 plants for each replicate and measured obtain their average shoot and root dry weight. The shoots and roots were separated by scissors and placed separately into the brown kraft paper bags, underwent the green removing process at 105 °C for 30 min, and were then dried at 75 °C in an oven around 3 days to constant weight.

### Determination of Soil Chemical Properties

We used the above four time-series coffee field samples for the soil chemical properties measurements. The soil pH and electrical conductivity (EC) were determined using a glass electrode pH meter (PHS-3C, INESA Scientific Instrument Co., Ltd, Shanghai, China) and a conductivity meter (DDS-307, INESA Scientific Instrument Co., Ltd, Shanghai, China) in a 1:5 soil water (w/v) suspension, respectively^16,41^. Organic matter and available N (hydrolytic nitrogen) were determined as reported by Tan *et al*.^28^. Soil available phosphorus (P) was extracted using a sodium hydrogen carbonate solution and then measured using the spectrophotometric method (U-3900H, HITACHI, Japan). Soil available potassium (K) was extracted with ammonium acetate and measured using flame photometry (HG-5, Yinze instrument equipment co. LTD, Shanghai, China)^48^. Other chemical properties including soil Ca, Mg, B, Fe, Mn, Cu and Zn were determined according to Bao^49^.

### Soil DNA Extraction and PCR Amplification

Soil DNA from the four time-series coffee field samples were extracted using the Power Soil DNA Isolation Kit (MoBio Laboratories Inc., Carlsbad, USA) according to the manufacturer’s instructions. The concentration and quality of the DNA were measured using a spectrophotometer (NanoDrop 2000, Wilmington, USA). The primer set 520 F (5′-AYTGGGYDTAAAGNG-3′) and 802 R (5′-TACNVGGGTATCTAATCC-3′)^50^ was used to amplify the V4 hypervariable region of the bacterial 16 S rRNA gene. ITS1F (5′-CTTGGTCATTTAGAGGAAGTAA-3′)^51^ and ITS2 (5′-GCTGCGTTCTTCATCGATGC-3′)^52^ were selected to target the fungal ITS1 region. Primer pairs were modified for sequencing by adding the forward Illumina Nextera adapter, a two basepair “linker” sequence, a unique 7-bp barcode sequence at the 5′ end of the forward primer, and the appropriate reverse Illumina Nextera adapter and linker sequence at the 5′ end of the reverse primer^18^. PCR was performed following previously published amplification conditions^14^. Briefly, 27 and 25 cycles were performed to amplify the fungal and bacterial templates, respectively. Then, the PCR products were purified using a PCR Purification Kit (Axygen Bio, USA) and pooled in equimolar concentrations of 10 ng/µl before sequencing. Finally, paired-end sequencing of the fungal and bacterial amplicons was carried out on the Illumina MiSeq sequencer at Personal Biotechnology Co., Ltd. (Shanghai, China).

### Amplicon sequencing Processing

Raw sequences were processed according to the previously established protocols^53^ with the following UPARSE pipeline^54^. Briefly, low-quality sequences with expected errors over 0.5 or a length shorter than 200 bp were discarded. After discarding singletons, the remaining reads were assigned to observed operational taxonomic units (OTUs) with an identity level threshold of 97% followed by the removal of putative chimeras using the UCHIME method^55^. After quality control, sequencing reads and coverage for each sample from the four time-series coffee fields were showed in Table S3. The coverage from bacterial and fungal samples was over 90% and 98% respectively, indicating that the depth of the sequencing reads were sufficient for our analysis. Finally, the bacterial sequences were matched against the Ribosomal Database Project (RDP) database^56^ and the fungal OTUs were classified using the UNITE database^57^ using the naïve Bayesian classifier implemented in Mothur with a threshold of 80% confidence.

### Sequence accession numbers

Sequences data (separate data of each sample) were deposited in the NCBI Sequence Read Archive (SRA) database under the BioProject: PRJNA374976 (the accession number SRR5262288 for bacteria and SRR5262289 for fungi).

### Statistical analysis

The number of observed OTUs (sobs), richness (Chao1 and ACE indexes)^58,59^ and diversity (Shannon index)^60^ were used to estimate the alpha diversity of the bacterial and fungal communities in each sample using the Mothur pipeline^61^. Rarefaction curves (allowing the calculation of species richness for a given number of individual samples) with the average number of observed OTUs were generated though Mothur^61^. In order to obtain an equivalent sequencing depth for the alpha diversity and rarefaction curves analysis, we rarefied each sample to the same depth (9146 sequences for bacterial and 11574 sequences for fungal data). We used the un-rarefied OTU table for all other analyses. Principal coordinate analysis (PCoA) based on Bray-Curtis distances was performed to explore the differences in bacterial and fungal community structure across all soil samples. Redundancy analysis (RDA) was performed in R (version 3.2.2) to examine the relationships among the time-series of coffee field samples, microbial community (abundant bacterial and fungal phyla) and soil variables (pH, EC, OM, Available N, P and K). Plant dry weight in pots, fresh weight of coffee fruit in fields, soil chemical characteristics, and bacterial and fungal taxa across the time-series of coffee field samples were compared using Duncan’s test in SPSS v 20.0 (SPSS Inc., USA). In addition, Pearson’s correlation relationships analysis was also tested in SPSS v 20.0 (SPSS Inc., USA).

## Electronic supplementary material

Supplementary Information
