# Supplementary material for: Long-Term Coffee Monoculture Alters Soil Chemical Properties and Microbial Communities
Source: Sci Rep. 2018 Apr 17;8:6116. doi: 10.1038/s41598-018-24537-2 (PMC5904153; doi:10.1038/s41598-018-24537-2)
Supplement: Supplementary file 1 — Supplementary Information [file 41598_2018_24537_MOESM1_ESM.pdf]

Title: Long-Term Coffee Monoculture Alters Soil Chemical Properties and Microbial Communities

Author list: Qingyun Zhao, Wu Xiong, Yizhang Xing, Yan Sun, Xingjun Lin, and Yunping Dong

Table S1 Soil fertilizer regimes in coffee cropping fields every year.

Time	Soil fertilizer regimes
May	Organic manure (cow manure) 4,500 kg ha^{-1};<br>KCl 150 kg ha^{-1};<br>Calcium superphosphate 150 kg ha^{-1};<br>Urea 150 kg ha^{-1};
July	KCl 150 kg ha^{-1};<br>Urea 150 kg ha^{-1};
October	KCl 150 kg ha^{-1};<br>Urea 150 kg ha^{-1};

Table S2 Fresh weight of coffee fruit in the 4 time-series coffee fields.

Coffee fields	Fruit fresh weight (kg/ha)
4Y	14026.50±1066.55 a
18Y	10361.25±1578.35 b
26Y	5083.88±1096.24 c
57Y	1829.25±113.22 d

Data were expressed as mean ± standard deviation (n = 3). The data in a column with a different letter differ significantly at Duncan's significance level 0.05.

"4Y", "18Y", "26Y", and "57Y" represent fields continuously cropped for 4, 18, 26, or 57 years, respectively.

Table S3 Bacterial and fungal sequencing reads and coverage for each sample from the four time-series coffee fields.

Treatments	**Bacterial** reads	**Bacterial** coverage	**Fungal** reads	**Fungal** coverage
4Y_1	44963	0.971	66095	0.996
4Y_2	40901	0.965	26106	0.990
4Y_3	37816	0.964	29064	0.990
4Y_4	42822	0.968	29047	0.991
18Y_1	38236	0.965	39214	0.993
18Y_2	29947	0.950	54509	0.995
18Y_3	28913	0.961	51972	0.995
18Y_4	38116	0.963	24571	0.990
26Y_1	42472	0.967	38085	0.994
26Y_2	44086	0.969	11574	0.980
26Y_3	30201	0.956	29632	0.992
26Y_4	24399	0.947	16749	0.987
57Y_1	52934	0.974	27966	0.991
57Y_2	40109	0.967	41394	0.994
57Y_3	20784	0.936	43956	0.995
57Y_4	9146	0.907	51884	0.995

"4Y", "18Y", "26Y", and "57Y" represent fields continuously cropped for 4, 18, 26, or 57 years, respectively.

Table S4 Pearson's correlation relationships between soil properties, bacterial and fungal abundant phyla (RA > 1%), abundant genera (top 20) and fresh weight of coffee fruit in the field.

**Soil properties**	Fresh weight of coffee fruit	**Bacterial and fungal phyla**	Fresh weight of coffee fruit	**Bacterial abundant genera**	Fresh weight of coffee fruit	**Fungal abundant genera**	Fresh weight of coffee fruit
**pH**	0.684	**Proteobacteria**	**0.902***	**Gp1**	-0.880	**Hypocrea**	-0.856
**EC**	-0.918*	**Acidobacteria**	**-0.783**	**Gp2**	-0.734	**Fusarium**	0.567
**OM**	0.832	**Bacteroidetes**	**0.957***	**Gp3**	-0.892	**Cryptococcus**	-0.915*
**N**	-0.752	**Verrucomicrobia**	**-0.260**	**Gp6**	0.942*	**Haematonectria**	0.660
**P**	-0.977*	**Actinobacteria**	**0.403**	**GpIIa**	0.423	**Xylogone**	-0.577
**K**	-0.050	**Firmicutes**	**-0.842**	**Gp4**	0.972*	**Trichosporon**	-0.429
**Ca**	0.327	**Chloroflexi**	**-0.892**	**Burkholderia**	-0.799	**Zopfiella**	0.065
**Mg**	-0.417	**Planctomycetes**	**-0.785**	**Gp13**	-0.975*	**Phaeosphaeria**	0.788
**B**	-0.474			**Terrimonas**	0.963*	**Coniochaeta**	0.842
**Fe**	-0.986**	**Ascomycota**	**0.895**	**Pseudomonas**	0.818	**Thermomyces**	0.826
**Mn**	-0.507	**Basidiomycota**	**-0.894**	**Nitrospira**	0.880	**Trichoderma**	0.880
**Cu**	-0.845			**Steroidobacter**	0.952*	**Staphylotrichum**	-0.785
**Zn**	-0.971*			**Thiobacillus**	-0.755	**Chaetomium**	0.890
				**Gemmatimonas**	0.982**	**Pyrenochaetopsis**	0.988**
				**Flavobacterium**	0.874	**Conlarium**	-0.920*
				**Bradyrhizobium**	0.700	**Aureobasidium**	-0.294
				**Geobacter**	-0.744	**Chloridium**	-0.304
				**Gp5**	0.697	**Phialemonium**	0.365
				**Sphingomonas**	0.745	**Dactylella**	0.877
				**Exiguobacterium**	0.585	**Gibberella**	0.734

RA is relative abundance. * represent significance at $P < 0.05$ and ** represent significance at $P < 0.01$.

Figure S1 Map for the sampling regime

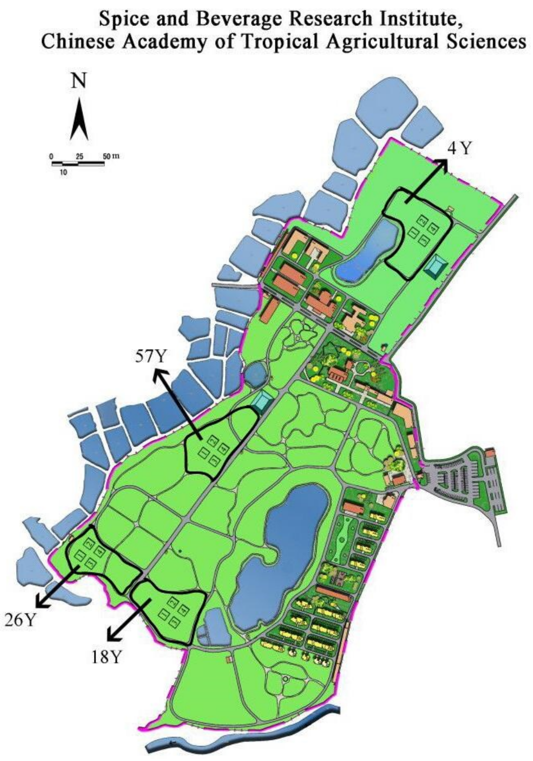

The experimental site is located at the Spice and Beverage Research Institute, Chinese Academy of Tropical Agricultural Sciences, Xinglong, Hainan province, China

“4Y”, “18Y”, “26Y”, and “57Y” represent fields continuously cropped for 4, 18, 26, or 57 years, respectively. “1”, “2”, “3”, and “4” represent the four subplots from each field.

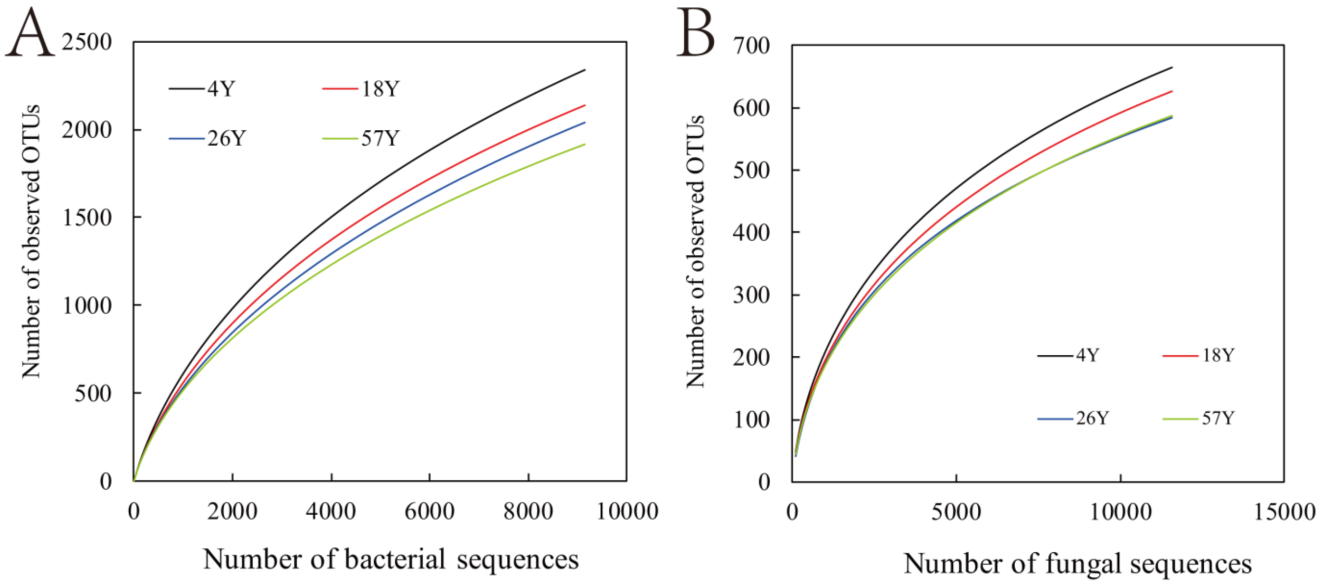

Figure S2 Rarefaction analysis at 3% dissimilarity levels (panel A: Bacteria; panel B: Fungi) for soil samples collected from coffee fields continuously cropped for 4 years (4Y), 18 years (18Y), 26 years (26Y) and 57 years (57Y).
